# Long peptic strictures of the esophagus due to reflux esophagitis: a case report

**DOI:** 10.1186/s40792-016-0190-1

**Published:** 2016-06-25

**Authors:** Yasushi Yamasaki, Soji Ozawa, Junya Oguma, Akihito Kazuno, Yamato Ninomiya

**Affiliations:** Department of Gastroenterological Surgery, Tokai University School of Medicine, 143 Shimokasuya, Isehara, Kanagawa 259-1193 Japan

**Keywords:** Reflux esophagitis, Stricture, Esophagectomy

## Abstract

**Background:**

Most of benign esophageal strictures caused by gastroesophageal reflux are short segments and can be treated by an endoscopic dilatation, but cases of long-segment stenosis requiring an esophagectomy are rare.

**Case presentation:**

A 62-year-old woman had undergone emergency surgery for a giant ovarian tumor rupture at another hospital. A duodenal perforation occurred after surgery but improved with conservative treatment. She had undergone long-term nasogastric tube placement for 4 months because she was on a mechanical ventilator and did not receive proton pump inhibitors (PPIs). Thereafter, the patient experienced dysphagia. An esophagogastroduodenoscopy (EGD) revealed circumferential reflux esophagitis (grade D) and a stricture located 25 to 40 cm from the incisor teeth. She received medical treatment with fasting and PPIs. The second EGD revealed that the reflux esophagitis had improved somewhat, but that the esophageal stricture had worsened. Thereafter, balloon dilatation was attempted, but the stricture did not improve and she was referred to our hospital. Finally, she was diagnosed as having a benign esophageal stricture caused by reflux esophagitis. She underwent a thoracoscopic esophagectomy with gastric tube reconstruction through the antethoracic route. Her postoperative course was uneventful. Pathologically, a circumferential stricture with white scar formation and no malignant cells were observed.

**Conclusions:**

We experienced a rare case requiring esophagectomy for long-segment stenosis caused by reflux esophagitis. It is suggested that the possibility of esophageal stricture needs to be kept in mind when treating GERD patients with long-term nasogastric tube placement.

## Background

Many esophageal strictures are malignant, and benign esophageal strictures are not common. Most of benign esophageal strictures caused by gastroesophageal reflux are short segments and can be treated by an endoscopic dilatation [1], but cases of long-segment stenosis requiring an esophagectomy are rare. Here, we report a case of long-segment stenosis associated with reflux esophagitis and long-term nasogastric tube placement.

## Case presentation

A 62-year-old woman had undergone emergency surgery for a giant ovarian tumor rupture at another hospital in December 2013. A duodenal perforation occurred after surgery but improved with conservative treatment. She had undergone long-term nasogastric tube placement for 4 months because she was on a mechanical ventilator and did not receive proton pump inhibitors (PPIs). Thereafter, the patient experienced dysphagia, and a video fluoroscopic examination of her swallowing revealed the reflux of contrast medium from the stomach to the esophagus in February 2014. An esophagogastroduodenoscopy (EGD) revealed circumferential reflux esophagitis (grade D) and a stricture located 25 to 40 cm from the incisor teeth. She received medical treatment with fasting and PPIs. She underwent an EGD again in March 2014. The reflux esophagitis had improved somewhat, but the esophageal stricture located 33 cm from the incisor teeth had worsened, making it difficult to pass the nasal endoscope. Thereafter, balloon dilatation was attempted, but the stricture did not improve and she was referred to our hospital in April 2014. She had a history of bronchial asthma.

Upon admission, she had a height of 152.5 cm, a weight of 41.6 kg, a body mass index of 17.8, a blood pressure of 108/58 mmHg, a temperature of 36.4 °C, and pulse rate of 74 beats/minute, with no significant physical findings. Laboratory findings showed a hemoglobin level of 11.9 g/dL, a serum glutamic oxaloacetic transaminase level of 54 U/L, and a serum glutamic pyruvic transaminase level of 119 U/L, indicating mild anemia and liver dysfunction.

**Fig. 1:**
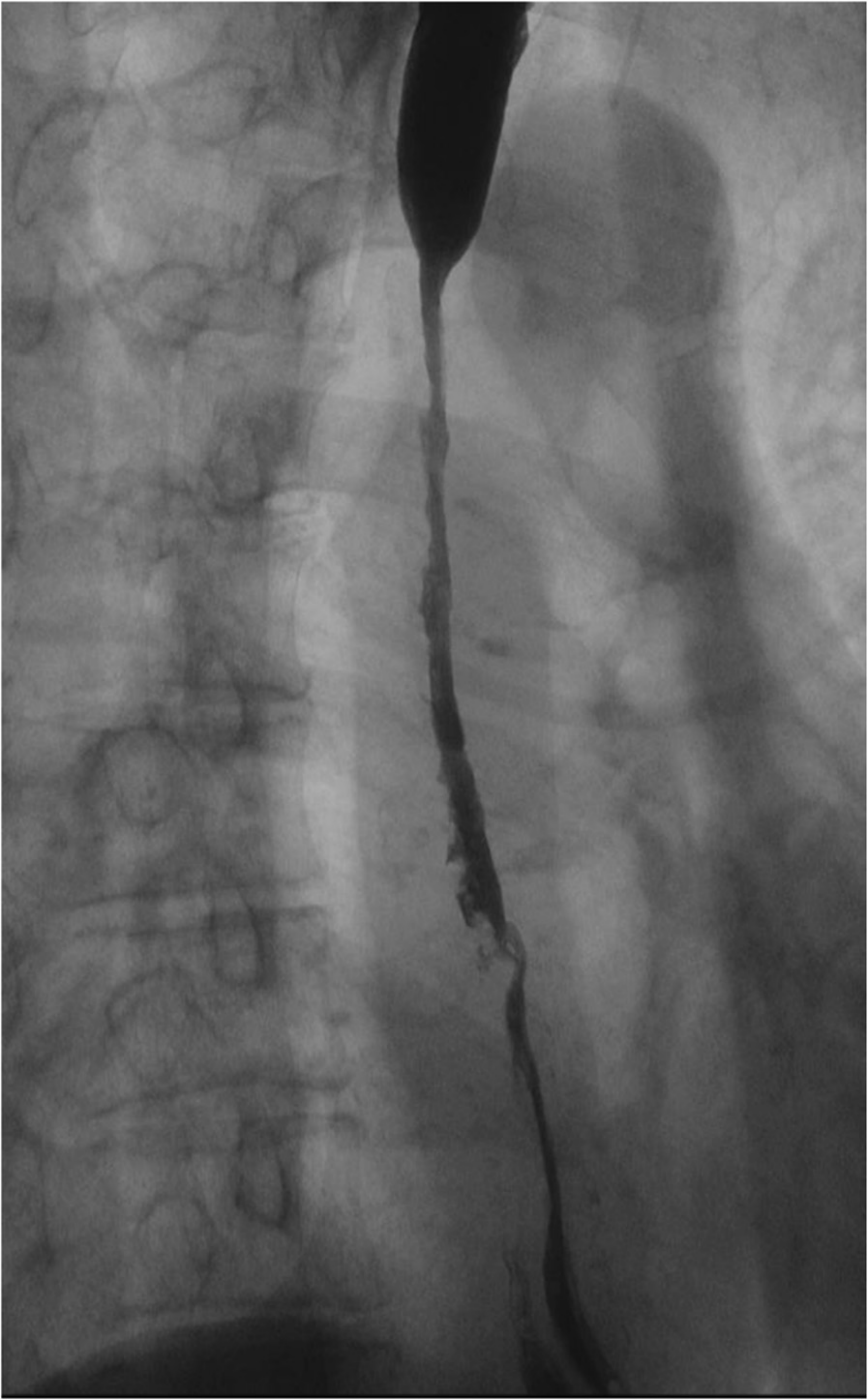
Upper gastrointestinal series. A severe stricture measuring 85 mm along the longitudinal axis was observed extending from the middle to lower thoracic esophagus

**Fig. 2:**
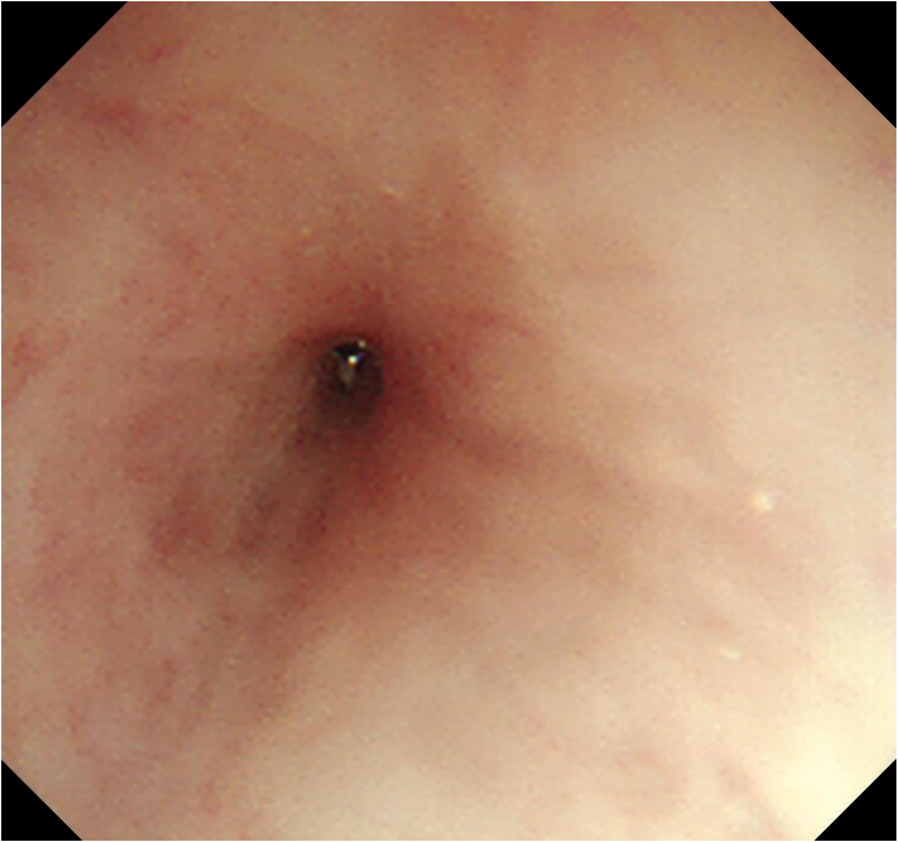
Esophagogastroduodenoscopy. A cicatricial stricture beginning 25 cm from the incisor teeth was observed. The stricture made it difficult to pass a small-diameter scope through the esophagus

**Fig. 3:**
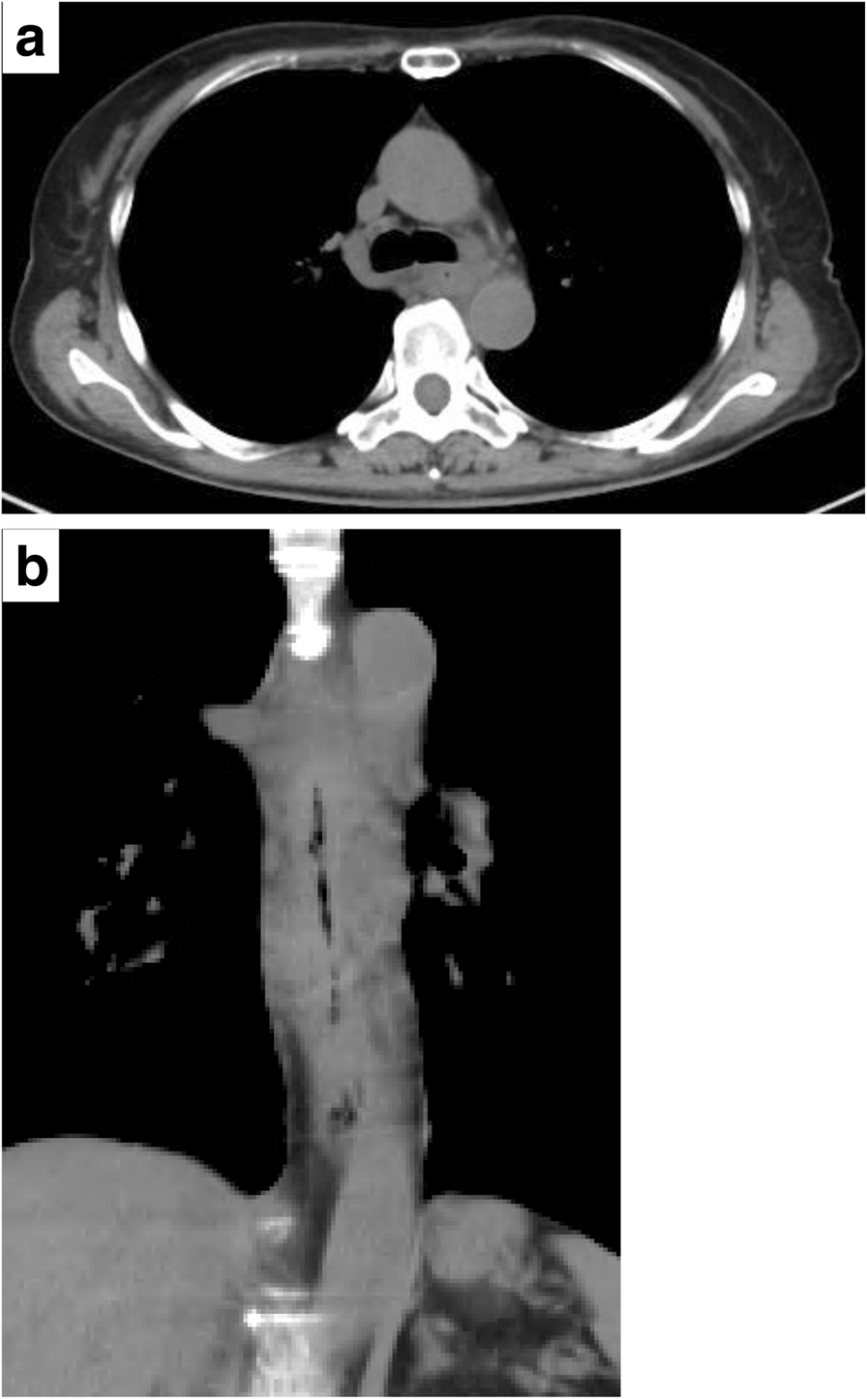
Preoperative CT. **a** Transverse image. **b** Coronal image: marked thickening of the esophageal wall was observed from the middle to lower thoracic esophagus

An upper gastrointestinal series revealed a severe stricture measuring 85 mm along the longitudinal axis from the middle to lower thoracic esophagus (Fig. 1). An EGD showed a cicatricial stricture beginning 25 cm from the incisor teeth, making it difficult to pass the endoscope through the esophagus (Fig. 2). A contrast-enhanced chest computed tomography (CT) scan examination revealed marked wall thickening from the middle to lower thoracic esophagus (Fig. 3). FDG-PET/CT showed a slight accumulation of isotope in the esophagus, and while a malignant disease could not be completely ruled out, a diagnosis of esophagitis seemed more probable. Although a 24-h pH monitoring test is necessary for the diagnosis of gastroesophageal reflux disease (GERD), this test was not performed because the pH catheter was not expected to pass through the esophagus because of the severe stricture. The clinical course and the above findings led to a diagnosis of benign esophageal stricture caused by reflux esophagitis.

**Fig. 4:**
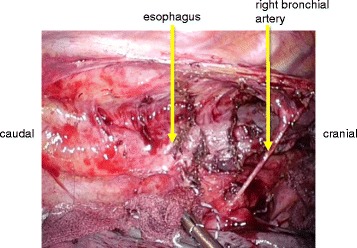
Findings during thoracoscopic mobilization of the middle and lower esophagus. A severe fibrotic change between the esophagus and the descending aorta was observed

Because endoscopic balloon dilatation did not improve the stricture, a thoracoscopic esophagectomy was performed [2]. The patients were placed in a prone position after intubation using a single-lumen endotracheal tube and a bronchial blocker tube. Only the left lung was ventilated, and a pneumothorax in the right chest was created using 6 mmHg of CO_2_ gas. Five trocars were inserted into the right thoracic cavity. The 12-mm trocar inserted into the ninth intercostal space on the inferior scapular angle line was used for a flexible endoscope. The anterior pleura and the posterior pleura of the upper posterior mediastinum were incised around the esophagus, and the upper esophagus was mobilized. Lymph node dissection was not performed because the patient had been diagnosed as having a benign esophageal stricture. The arch of the azygos vein was divided, and the right bronchial artery was preserved. Next, the anterior pleura and the posterior pleura of the middle and lower posterior mediastinum were incised around the esophagus. When the middle and lower esophagus was also mobilized, a severe fibrotic change between the esophagus and the descending aorta was observed. This fibrotic change seemed to be similar to the change in a case that had received chemoradiation therapy (Fig. 4). Finally, the upper esophagus was transected using a stapler device, and the thoracic esophagus was successfully mobilized. At the end of the thoracoscopic procedure, a single 32-Fr chest tube was inserted. The patient was then placed in a supine position. The stomach was mobilized, and a gastric tube was created. An antethoracic route was chosen because the patient had a BMI of 17.8 kg/m^2^ and was treated with total parenteral nutrition, which posed a surgical risk. An anastomosis between the cervical esophagus and the gastric tube was performed using a circular stapler in the neck, and a tube jejunostomy was created.

**Fig. 5:**
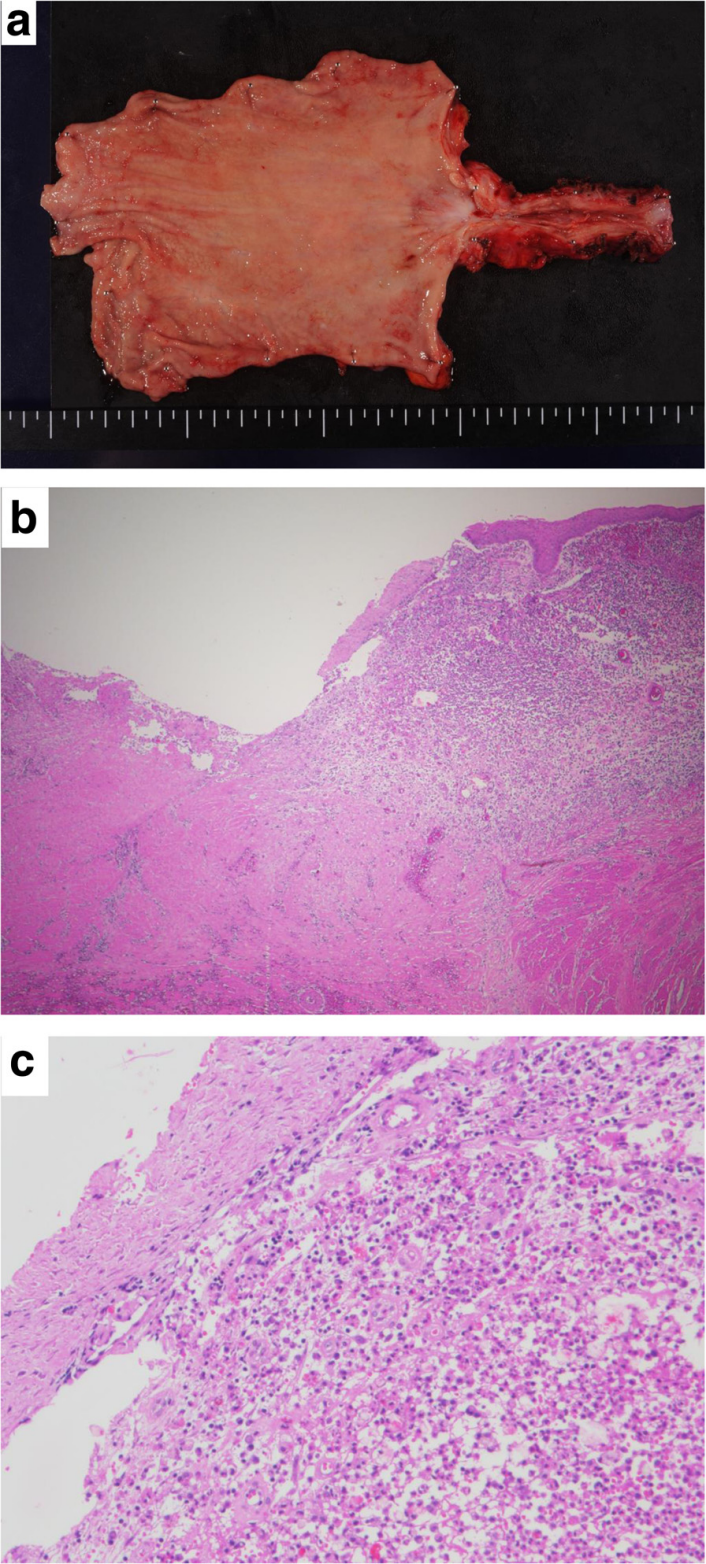
Gross and microscopic findings. **a** Macroscopically, a long segment of circumferential thickening of the esophageal wall was observed. **b** Erosion and ulceration were visible (HE staining, ×20). **c** Inflammatory cell infiltration was observed. No malignant findings were seen (HE staining, ×100)

The gross pathological findings showed a circumferential stricture with white scar formation from the lower esophagus to the cardia. Histologically, the infiltration of inflammatory cells, mainly neutrophils, lymphocytes and plasma cells, and fibrosis were observed. Erosion and ulceration were seen, but there were no malignant findings (Fig. 5).

Her postoperative course was uneventful, and she was discharged on day 19. At present, 1 year and 8 months after surgery, she is attending an outpatient clinic.

### Discussion

Esophageal strictures have a variety of causes. Makuuchi classified esophageal strictures into functional and organic strictures. The former included esophageal achalasia, diffuse spasm, vigorous achalasia, and nutcracker esophagus, while the latter included malignant neoplasms, reflux esophagitis, esophageal web, and postoperative anastomotic stricture. It was also described that most esophageal strictures caused by reflux esophagitis occur at the lower end of the esophagus [3]. The majority of esophageal strictures are malignant, and benign esophageal strictures are rare. Recently, the use of PPIs has decreased the number of patients with benign esophageal strictures. The incidence of esophageal stricture is 1.1 among 10,000 persons, and 68 % of esophageal stricture cases are peptic. GERD, hernia, ulcer, and heavy drinking increase the risk of stricture [4]. Also, patients with esophagitis reportedly have an eight times higher risk of stricture [5].

The mechanism of stricture formation arising from reflux esophagitis is considered to be as follows: gastric acid reflux causes inflammation in the lamina propria which is then disrupted, leading to stricture formation [6]. In addition, esophageal stricture caused by long-term nasogastric tube placement has also been reported, and according to the report, the use of a nasogastric tube for more than 2–4 weeks should be avoided [7]. The present patient had reflux esophagitis, had undergone long-term nasogastric tube placement, and did not receive PPIs, placing her at a high risk for stricture.

Malignant transformation has to be taken into account in esophageal strictures associated with reflux esophagitis. The reflux of digestive juice is widely known to cause the development of Barrett’s esophagus, eventually leading to Barrett’s esophageal cancer, and controlling the reflux of digestive juice to prevent malignant transformation is of primary importance. In this case, the clinical course and the findings of CT and FDG-PET/CT led to a diagnosis of benign esophageal stricture caused by reflux esophagitis. Postoperatively, the resected specimen revealed no malignant findings and the preoperative diagnosis was proven to be correct.

van Boeckel and Siersema previously described an algorithm for treatment selection. According to their report, endoscopic dilatation is successful in more than 80–90 % of cases, but recurrence occurs within 1 year in one third of cases. At the time of recurrence, the combination of dilatation and steroid injection or stenting should be considered. Surgery should be a last-resort treatment option [8].

Surgery for benign esophageal stricture can be classified into two types. In cases where endoscopic dilatation can be performed, antireflux surgery, such as a Toupet, Belsey, or Nissen procedure, is performed following stricture dilatation. In cases where the stricture cannot be dilated, an esophagectomy and reconstruction are performed. In addition, surgery is indicated in (1) patients in whom stricture dilatation is not useful, (2) patients with frequent aphagia, (3) patients with intractable refractory esophagitis, and (4) patients with aspiration pneumonia, etc. [9]. In the present case, surgery was performed because the patient was refractory to endoscopic treatment and had reflux esophagitis (grade D), and the extended length of the stricture persisted even after conservative treatment.

The present case was characterized by an 85-mm-long segment of stenosis extending from the middle to lower thoracic esophagus. As described above, most strictures associated with reflux esophagitis are usually localized at the lower end of the lower esophagus and are found at a distance of 1 to 4 cm from the gastroesophageal junction [10, 11]. A search of PubMed using the keywords “esophagus,” “stricture,” and “benign” yielded five articles on benign stricture caused by the reflux of digestive juice describing the length of the stricture [12–16] (Table 1). None of the severe strictures measured >8 cm in length in these reports. Accordingly, the present case appears to be relatively rare.

**Table 1:** Reports of benign stricture caused by the gastroesophageal reflux

No.	Author (year)	*n*	Age (years old) (mean)	Sex (male:female)	Mean length of stricture (mm)	Cause of stricture	Treatment
1	Rudstrom P (1954) [12]	2	49.5	2:0	45	Peptic stricture	2: surgical
2	Raptis S (1972) [13]	100	69	30:70	10–70(range)	Peptic stricture (most cases)	50: surgical
							50: non-surgical
3	Jaffray B (1998) [14]	113	67	63:50	20–50(range)	Peptic stricture	13: surgical
							100: non-surgical
4	Braghetto I (2002) [15]	185	64.1	135:50	25.1 ± 11	Peptic stricture	170: surgical
							15: non-surgical
5	Qureshi S (2010) [16]	10	10	3:7	39 ± 17	Peptic stricture	Not described
Present case	Yamasaki Y (2015)	1	62	0:1	85	Peptic stricture	1: surgical

## Conclusions

We experienced a case requiring esophagectomy for long-segment stenosis caused by reflux esophagitis. It is suggested that the possibility of esophageal stricture needs to be kept in mind when treating GERD patients with long-term nasogastric tube placement.

## Consent for publication

Written informed consent was obtained from the patient for publication of this case report and any accompanying images.

## Abbreviations

CT, computed tomography; GERD, gastroesophageal reflux disease; PPIs, proton pump inhibitors.
